# Supplementary material for: Dichotomy of the BSL phosphatase signaling spatially regulates MAPK components in stomatal fate determination
Source: Nat Commun. 2022 May 4;13:2438. doi: 10.1038/s41467-022-30254-2 (PMC9068801; doi:10.1038/s41467-022-30254-2)
Supplement: Supplementary file 1 — Supplementary Information [file 41467_2022_30254_MOESM1_ESM.pdf]

Supplementary Information for

Dichotomy of the BSL phosphatase signaling spatially regulates MAPK components in stomatal fate determination

Xiaoyu Guo[1] , Xue Ding[1], and Juan Dong[1,2,*]

Correspondence to: Juan Dong (dong@waksman.rutgers.edu)

This file includes:

Supplementary Figures 1-8

Supplementary Table 1

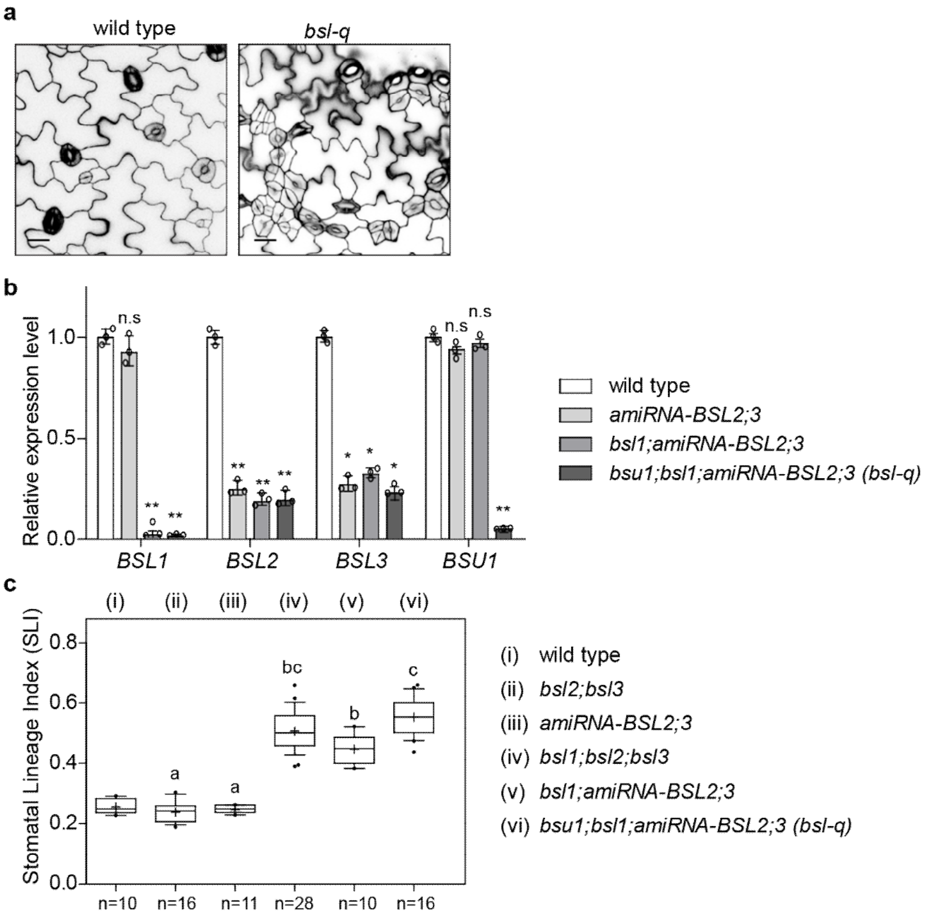

Supplementary Fig. 1 | Characterization of mutants containing *amiR-BSL2;3* (*microRNA*-induced knockdown of *BSL2* and *BSL3*)

a, Representative confocal images show five-day adaxial cotyledon epidermis of the wild-type and *bsl-q* mutant. Note, large stomatal patches in *bsl-q* were observed at the leaf edges. Cell walls were stained with propidium iodide (PI), and images were converted to black/white. Scale bar, 20 µm.

b, Quantitative PCR (qPCR) data show relative expression levels of *BSL1*, *BSL2*, *BSL3*, and *BSU1* in indicated genetic backgrounds. Total RNAs were extracted from 3-day-old seedlings. Gene expression levels were normalized by *ACTIN2* and relative expression levels of *BSL* were compared with the values in the wild type. Experiments were independently repeated three times. Data are presented as mean ± SD. Statistical analysis was performed with one-way ANOVA and Tukey's post hoc test. n.s., not significant; ** $P < 0.005$ and * $P < 0.05$. Exact P values obtained by unpaired t-test are 0.8807, 0.0011 and 0.001 for BSL1, 0.0024, 0.002 and

0.0022 for BSL2, 0.0075, 0.0082 and 0.0056 for BSL3, 0.4341, 0.659 and 0.0032 for BSU1. Primers used were listed in Supplementary Table 1.

c, Quantification of stomatal lineage index of the designated genotypes. Box plot shows first and third quartiles, median (line) and mean (cross). Letters indicate one-way ANOVA; Tukey's test ($P < 0.01$). P values are 0.1384 for i *vs*. ii, 0.3308 for i *vs*. iii, 5.4086^{e-19} for i *vs*. iv, 5.0942^{e-8} for i *vs*. v, and 1.8482^{e-10} for i *vs*. vi, obtained by unpaired t-test. n, number of cotyledons used for quantification of each genetic background.

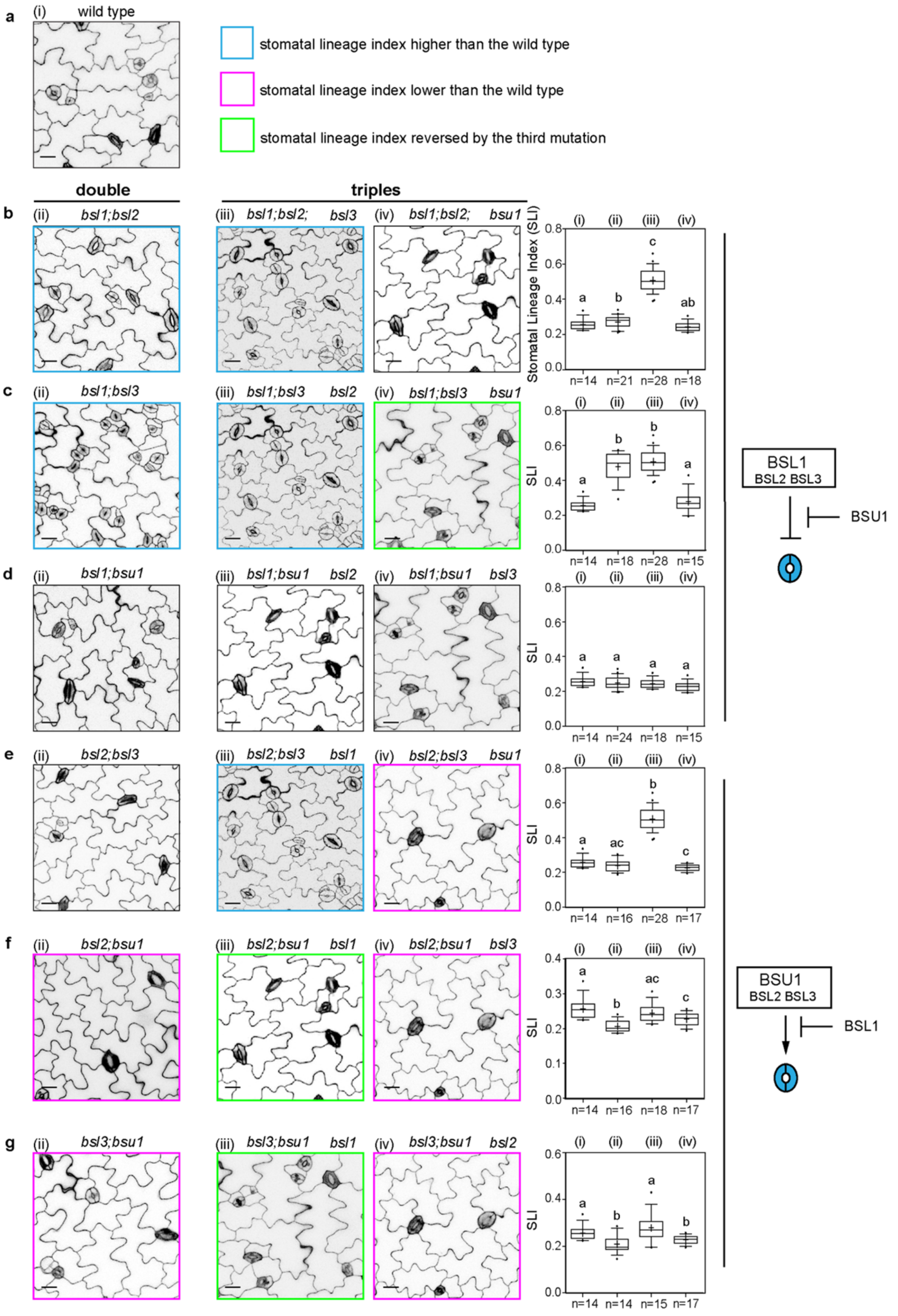

a
(i) wild type
stomatal lineage index higher than the wild type
stomatal lineage index lower than the wild type
stomatal lineage index reversed by the third mutation
double
triples
b
(ii) bsl1;bsl2
(iii) bsl1;bsl2; bsl3
(iv) bsl1;bsl2; bsu1
Stomatal Lineage Index (SLI)
n=14 n=21 n=28 n=18
c
(ii) bsl1;bsl3
(iii) bsl1;bsl3 bsl2
(iv) bsl1;bsl3 bsu1
n=14 n=18 n=28 n=15
BSL1
BSL2 BSL3
BSU1
d
(ii) bsl1;bsu1
(iii) bsl1;bsu1 bsl2
(iv) bsl1;bsu1 bsl3
n=14 n=24 n=18 n=15
e
(ii) bsl2;bsl3
(iii) bsl2;bsl3 bsl1
(iv) bsl2;bsl3 bsu1
n=14 n=16 n=28 n=17
f
(ii) bsl2;bsu1
(iii) bsl2;bsu1 bsl1
(iv) bsl2;bsu1 bsl3
n=14 n=16 n=18 n=17
BSU1
BSL2 BSL3
BSL1
g
(ii) bsl3;bsu1
(iii) bsl3;bsu1 bsl1
(iv) bsl3;bsu1 bsl2
SLI
n=14 n=14 n=15 n=17

Supplementary Fig. 2 | Stomatal phenotypes in *bsl* mutants

a-g, Confocal images of 5-dpg adaxial side of cotyledon epidermis for indicated genotypes. Cell walls were stained with propidium iodide (PI), and images were converted to black/white. Representative images for each genotype were selected from at least five samples. Blue and pink boxes highlight genotypes displaying elevated and reduced stomatal lineage index (SLI), respectively. Green boxes highlight genotypes with SLI reversed by the third mutation. Scale bar, 20 µm. Box plots on each row show quantification of stomatal lineage index of the designated genotypes on the left. Box plot shows first and third quartiles, median (line) and mean (cross). Letters indicate statistically significant differences between stomatal lineage index based on one-way ANOVA; Tukey's test ($P < 0.01$). Exact P values are 7.58e-10 for *bsl1;bsl3*, 7.569e-20 for *bsl1;bsl2;bsl3*, 0.2435 for *bsl1;bsl3;bsu1*, 1.77718e-05 for *bsl2;bsu1*, 0.0045948 for *bsl2;bsl3;bsu1*, 0.2213566 for *bsl1;bsl2;bsu1*, 0.1517853 for *bsl1;bsl2*, 0.5149783 for *bsl1;bsu1,* 0.1438238 for *bsl2;bsl3*, and 0.000772 for *bsl3;bsu1*, by unpaired t-test. N, number of cotyledons used for quantification of each genotype. Graphics on the right describe genetic regulation of BSL genes in stomatal production. Note, T-bars indicate negative regulations, and arrows indicate positive regulations in stomatal development.

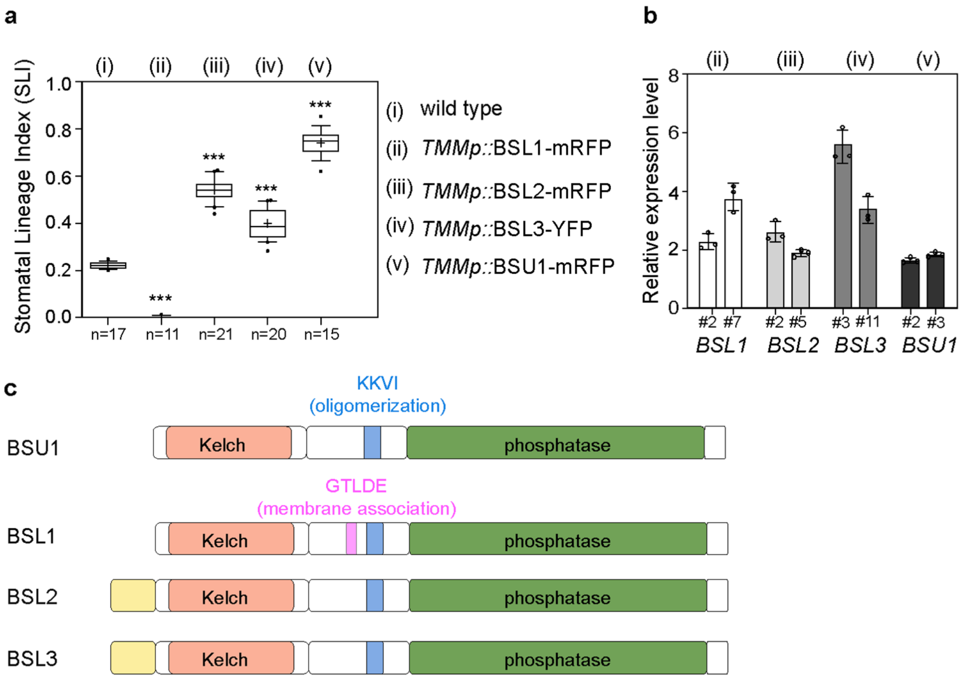

Supplementary Fig. 3 | Differential regulation of BSL proteins in stomatal development

A, Quantification of stomatal lineage index in wild type plants and overexpression of *BSL* genes in the stomatal lineage (driven by the *TMM* promoter). Box plots show the first and third quartiles, split by the median (line) and mean (cross). N, number of biologically independent cotyledons. Statistical analysis was performed with one-way ANOVA and Tukey's post hoc test. Values were compared to those of the wild type. ****P* < 0.0001. *P* values obtained by unpaired t-test are 3.32006^{e-25} for ii *vs*. i, 1.39825^{-19} for iii *vs*. i, 3.16333^{e-11} for iv *vs*. i, and 1.13742^{e-16} for v *vs*. i,.

b, qPCR data show relative expression levels of each *BSL* gene in transgenic plants used in **a** and Fig.**2a**. Total RNAs were extracted from 3-day-old seedlings. Gene expression levels were normalized by *ACTIN2* and relative expression levels of *BSL* were compared with the values in the wild type. Experiments were independently repeated three times. Data are presented as mean ± SD. Primers used were listed in Supplementary Table 1.

c, Diagrams depict domain structures of the BSL proteins in *Arabidopsis*. The highly conserved core catalytic domains are depicted in green, and the Kelch-like domains are shown in light red.

Blue boxes mark conserved amino acids (KKVI motif) required for BSL protein oligomerization.

A unique GTLDE domain (pink box) mediates membrane localization of BSL1.

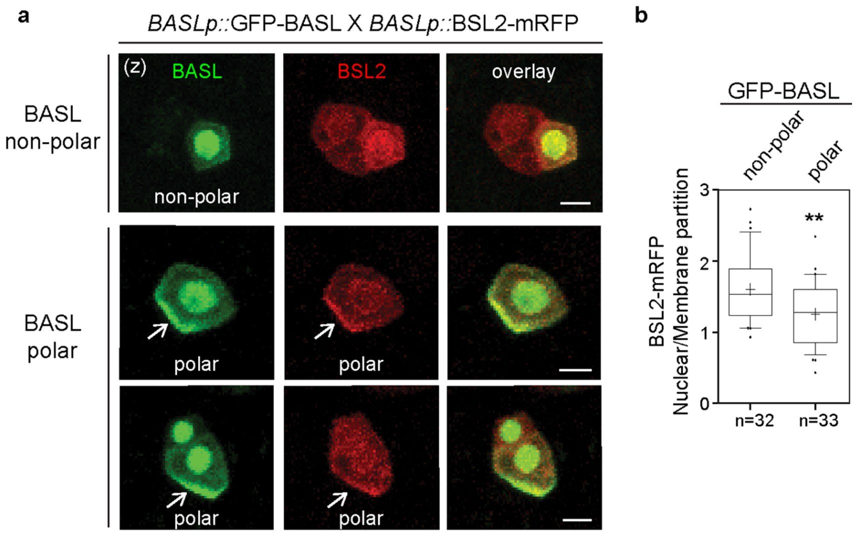

Supplementary Fig. 4 | Differential nuclear/membrane partition of BSL2 associated with BASL polarization

a, Representative confocal images show protein subcellular localization of BSL2 (red) co-expressed with BASL (green). BASL polarization in stomatal lineage cells is marked with white arrow. Data represent results of three independent experiments. (z), images are z-staked. Scale bar, 5 µm.

b. Quantification of nuclear/membrane (N/M) partition of BSL2–mRFP in cells co-expressing GFP-BASL without or with BASL polarization. Box plot shows first and third quartiles, median (line) and mean (cross). Two-tailed Student's t-test. n, number of cells expressing both GFP-BASL and BSL2-mRFP. $**P = 0.002496$.

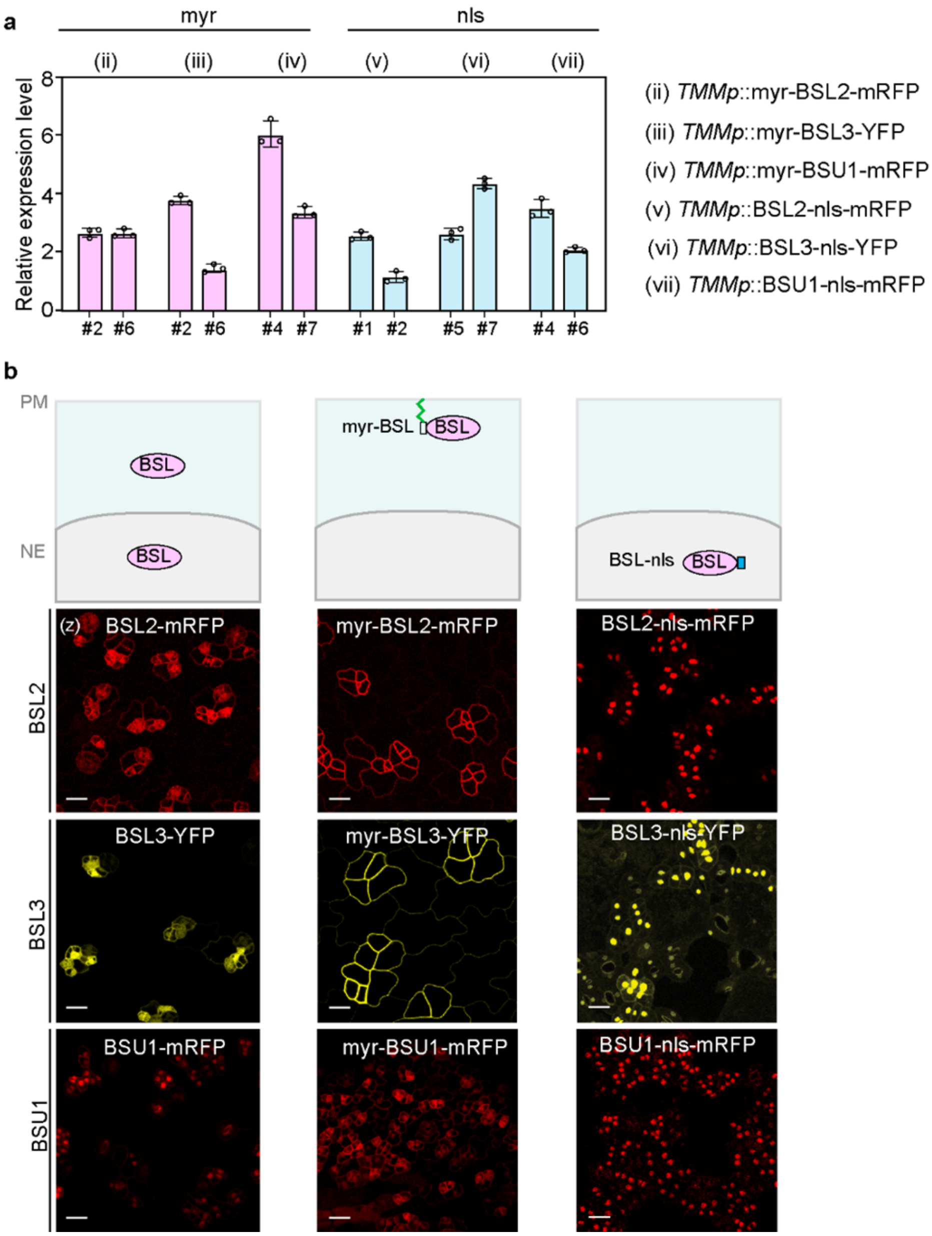

Supplementary Fig. 5 | Spatial overexpression of BSL genes at the subcellular level in stomatal lineage cells

a, qPCR results evaluate relative expression levels of *BSL2*, *BSL3* or *BSU1* in stomatal lineage-specific overexpression plants (all driven by the *TMM* promoter). Total RNAs were extracted from 3-day-old transgenic seedlings grown on solid ½ MS. The expression levels of the transgene were normalized to that of *ACTIN2*, and the values for each genotype were compared to that of the wild type. All experiments were repeated three times independently. Histograms represent mean ± SD. Primers used in the qPCR experiments listed in Supplementary Table 1.

b, Diagram and confocal images show overall subcellular localization of indicated proteins in 5-dpg adaxial side of cotyledon epidermis. myr, BSL proteins tagged with the myristoylation site; nls, BSL proteins tagged with nuclear localization sequence. Data represent results of three independent experiments. (z), all images are z-staked. Scale bar, 20 μm.

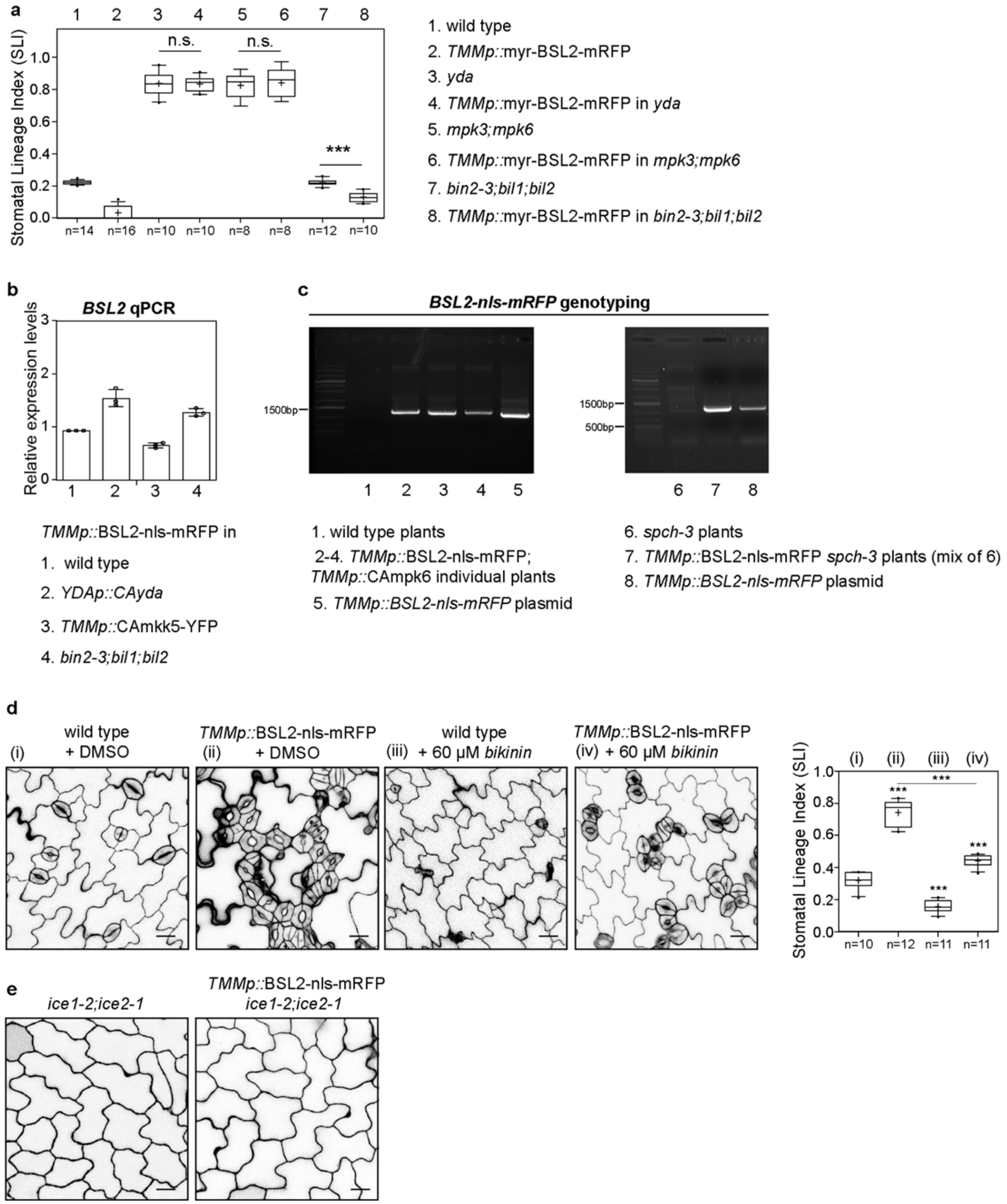

Supplementary Fig. 6 | Genetic positioning of BSL2 function in the signaling pathways regulating stomatal development

a, Quantification of stomatal lineage index of the genotypes shown in Fig. **4b-e**. Box plot shows first and third quartiles, median (line) and mean (cross). n, number of biologically independent cotyledons. One-way ANOVA with Tukey's post hoc test were used for comparisons as indicated. n.s., not significant. *** $P < 0.0001$. Exact P values by unpaired t-test are 0.944102 (3 *vs*. 4), 0.749086 (5 *vs*. 6), and 1.28988e-06 (7 *vs*. 8), respectively.

b, qPCR data show relative expression levels of *BSL2* in the indicated genotypes. Total RNAs were extracted from 3-day-old seedlings. Gene expression levels were normalized by *ACTIN2* and relative expression levels of *BSL2* were compared with the values of *TMMp*::BSL2-nls-mRFP in the wild type plants. Experiments were independently repeated three times. Data are presented as mean ± SD. Primers used were listed in Supplementary Table 1.

c, The presence of transgene (*TMMp::*BSL2-nls-mRFP) was examined by PCR-based genotyping. The expression of *CAmpk6* or loss-of-function *spch* mutation lead to the absence of stomatal lineage cells, disallowing the evaluation of the transcripts of the transgene *TMMp::*BSL2-nls-mRFP. n, sample number of three biologically independent experiments. Primers used were listed in Supplementary Table 1.

d, Confocal images of abaxial cotyledon epidermis of 5-dpg wild type or *TMMp*::BSL2-nls-mRFP seedlings treated with DMSO (control) or with 60 μM *bikinin*. Right, Quantification of the stomatal lineage index of the genotypes shown on the left. n, number of biologically independent cotyledons. Box plots show the first and third quartiles, split by the median (line) and mean (cross). Statistical analysis was performed with one-way ANOVA and Tukey's post hoc test. ***$P < 0.0001$. Exact P values obtained by unpaired t-test are 5.61^{e-12} for i *vs*. ii, 3.29564^{e-7} for i *vs*. iii, 1.35383^{e-5} for i *vs*. iv, 3.10239^{e-9} for ii *vs*. iv.

e, Confocal images of 5-dpg seedlings of *ice1-2;ice2-1* double mutant (left) and *TMMp*::BSL2-nls-mRFP in *ice1-2;ice2-1* (right). For images in **d** and **e**, cell outlines were stained with propidium iodide, and confocal images were converted to black/white. Representative confocal images were selected from at least three biologically independent cotyledons. Scale bar, 20 μm.

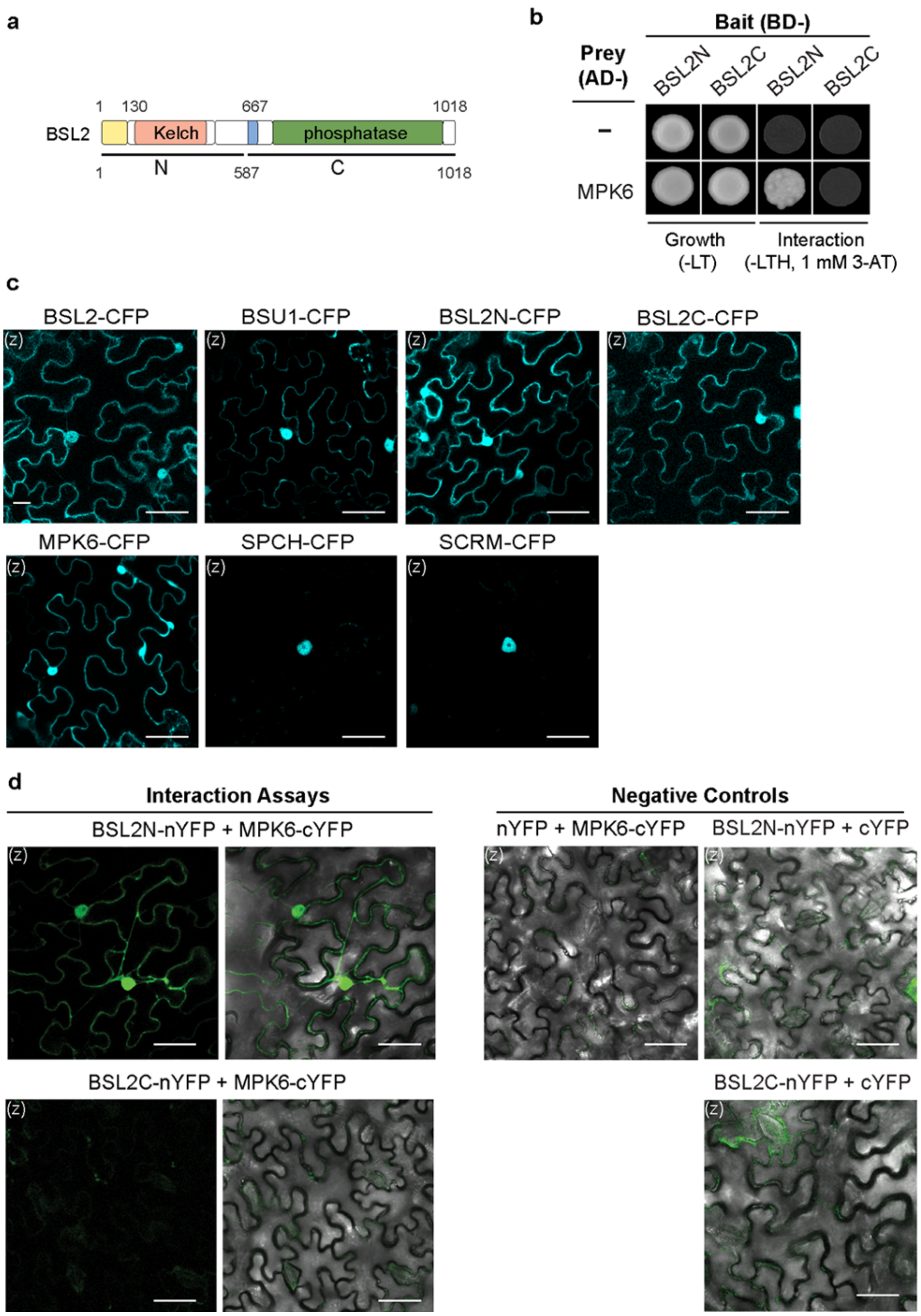

Supplementary Fig. 7 | The N-terminal region of BSL2 mediates interaction with MPK6

a, Domain structure of the BSL2 proteins in *Arabidopsis*. BSL2N and BSL2C indicate the N-terminus and C-terminus of BSL2, respectively.

b, Results of yeast two-hybrid assay show the N-terminal of BSL2 is sufficient to interact with MPK6, while no interactions between C-terminal of BSL2 and MPK6 was identified. Bait, “BD-” indicates Gal4 DNA-binding domain; Prey, “AD-” indicates Gal4 activation domain. “Growth

controls”, assays performed using rich media (-Leu-Trp); “Interaction tests”, assays performed using synthetic dropout medium (-Leu-Trp-His; 1 mM 3-AT added to suppress bait auto-activation).

c, Representative confocal images show subcellular localization of indicated proteins (tagged with CFP) in *N. benthamiana* leaf epidermal cells.

d, Results of bimolecular fluorescence complementation (BiFC) assays in *N. benthamiana* leaf epidermal cells show N-terminal of BSL2 interacts with MPK6 in plant cells. nYFP, N-terminal YFP; cYFP, C-terminal YFP. YFP signals indicate protein-protein interactions. For **c** and **d**, Data represent results of three independent experiments. Scale bar, 50 µm. All images are z-staked (z).

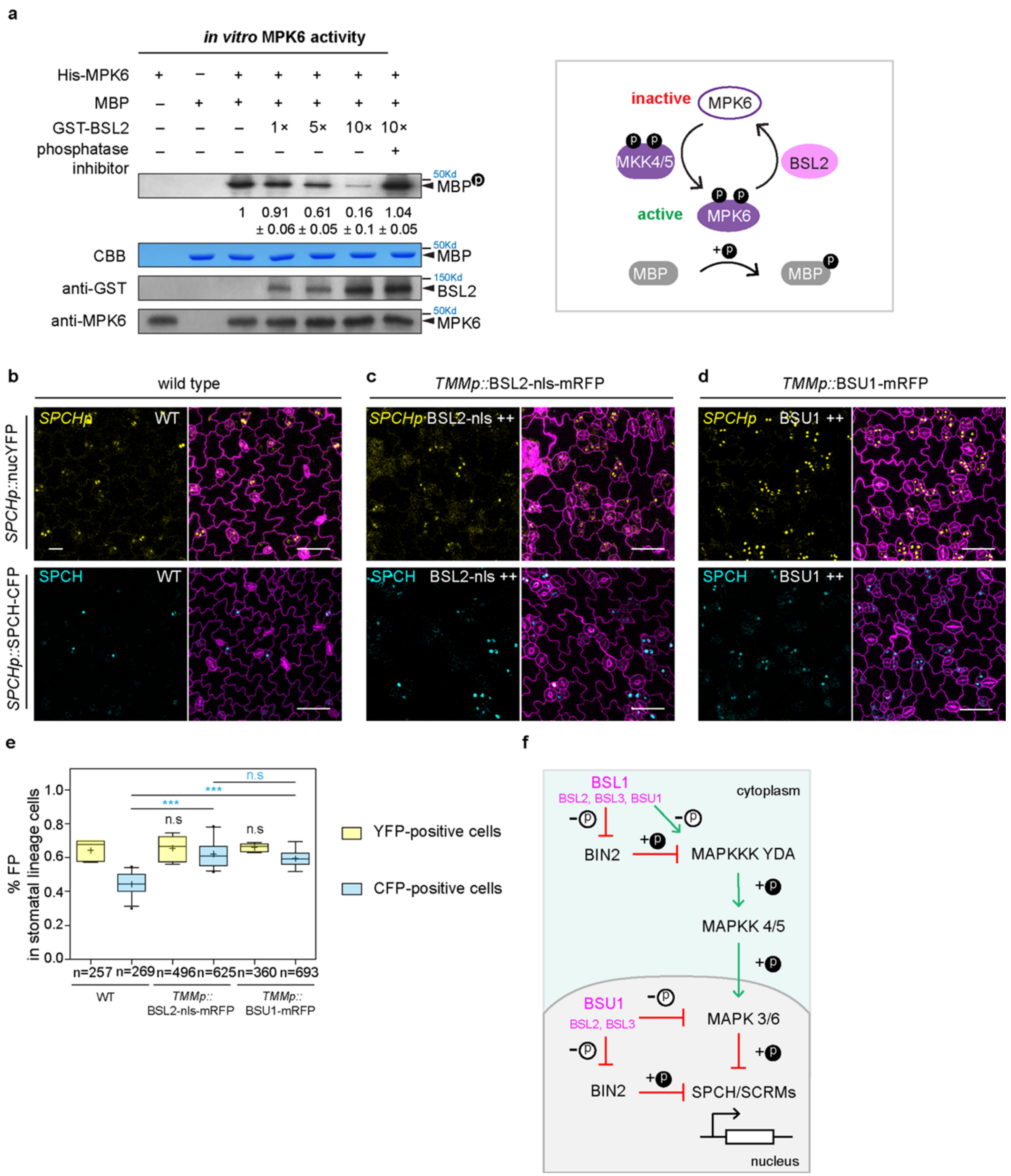

Supplementary Fig. 8 | Nuclear BSU1 and BSL2-nls promote protein stability of the transcription factor SPCH

a, Kinase activity of the recombinant MPK6 protein in the presence or absence of BSL2 was examined using myelin basic protein (MBP) as an *in vitro* substrate (Left). MPK6 and BSL2

were fused with a histidine-tag (His) and glutathione S-transferase (GST), respectively. Thio-phosphorylated MBP was alkylated and detected by immunoblot using anti-alkylated thiophosphate antibody. Protein levels of His-MPK6 or GST-BSL2 were examined using anti-MPK6 or anti-GST antibody respectively. Numbers indicate relative amount of phosphorylated MBP protein of three biological replicates. Data are presented as mean ± SD. CBB: Coomassie Brilliant Blue. Right: Schematic depicts the kinase activity of MPK6 in phosphorylating the MBP substrate can be positively regulated by MKK4/5-mediated phosphorylation and negatively regulated by BSL2-mediated dephosphorylation.

b-d, Confocal images (all z-projections) of 3-dpg seedlings expressing transcriptional fusion *SPCHp*::nucYFP (yellow) or translational fusion *SPCHp*::SPCH-CFP (cyan) in the wild type background (**b**), in *TMMp*::BSL2-nls-mRFP (**c**), or in *TMMp*::BSU1-mRFP (**d**). Cell outlines were visualized by PI staining (magenta). Data represent results of three independent experiments. Scale, 50 μm.

e, Ratios of YFP- or CFP-positive cells relative to total stomatal lineage cells in 3-dpg seedlings in (**b-d**). n, total number of stomatal lineage cells collected from 5 to 10 cotyledons. Box plots show the first and third quartiles, split by the median (line) and mean (cross). Statistical analysis was performed with one-way ANOVA and Tukey's post hoc test. n.s., not significant. ***P < 0.0001. Black font indicates the comparison with YFP-positive cells in wild type. Exact P values obtained by unpaired t-test are 0.7322 and 0.5018, respectively. Blue font indicates the pairwise comparisons as specified. Exact P values are 5.21072^{e-5}, 0.000153, and 0.4159, respectively.

f, A more elaborated working model: A BSL phosphatases-based signaling dichotomy compartmentalizes key signaling events to control stomatal development in *Arabidopsis*. At the cell cortex close to the PM, BSL1 is a predominant regulator, together with the other three BSL phosphatases, activating the MAPKKK YODA to promote MAPK signaling. In addition, the BSL proteins promote the dissociation of the BIN2 kinases from the PM to release the BIN2-

mediated inhibition on YDA, allowing YDA to be further activated. Downstream YDA, activated MPK3/6 molecules phosphorylate the key stomatal fate transcription factors SPCH and ICE1/SCRMs for degradation, thereby suppressing stomatal production. By strikingly contrast, in the nucleus, BSU1 plays a primary role, together with BSL2 and BSL3, deactivating MPK3/6, resulting in stabilized SPCH and ICE1/SCRMs, thereby promoting stomatal production. In addition, the inhibition of BIN2 on SPCH can be alleviated by the nuclear function of BSL2/BSL3/BSU1 phosphatases, thereby allowing SPCH to be further stabilized to promote stomatal production in *Arabidopsis*.

Supplementary Table

Table S1. List of primers used in this study. F, forward primer; R, reverse primer.

**Purpose**	**Primer name**	**Sequence (5'-3')**
Genotyping	SALK_051383(BSL1) LP	TGATTAAATCTTGTCCACGCC
	SALK_051383(BSL1) RP	GCTTCATCCGAGAGCTGTATG
	SALK_055335(BSL2) LP	CATTAGCAAAGTTCTGCCAGC
	SALK_055335(BSL2) RP	GTTCCAGAGCAGATGGAGATG
	SALK_072437(BSL3) LP	CCTGCAAAATATCAATGCTTAG
	SALK_072437(BSL3) RP	TAATGCACTTTTTGGTTTCCG
	SALK_030721(BSU1) LP	ACGTTCCACTTCAACATGGAG
	SALK_030721(BSU1) RP	TCTTTAACCATGCTTCGAACC
	BSL2-C-F	CGTGGTTGGAAGCCTCCTGTTC
	mRFP-Cseq	CACGCGCTCCCACTTGAAG
qPCR	BSU1-qPCR-R	GTTGGCGACGGCAGTAGTACTG
	BSL1-qPCR-R	CCTCCCTCAATAGCGGTGGCG
	BSL2-qPCR-R	GCTCTTGTCCAACAACCGCA
	BSL3-qPCR-R	CTGTTGCTGCTGTTGTTG
Cloning	BSL1-F-NotI	GCTCCGCGGCCGCC ATGGGCTCGAAGCCTTG
	BSL1-R-AscI	A GGCGCGCCC GATGTATGCAAGCGAGCTTCTG
	BSL2-F-NotI	GCTCCGCGGCCGCC ATGGATGAAGATTCGTCTATGG
	BSL2-R-AscI	A GGCGCGCCC CATCCAAGCCAGAGAACC
	BSL3-F-NotI	GCTCCGCGGCCGCC ATGGATTTGGATTCTTCAATG
	BSL3-R-AscI	A GGCGCGCCC TATCCAAGCAAGAGAGC
	BSU1-F-NotI	GCTCCGCGGCCGCC ATGGCTCCTGATCAATCTTATC
	BSU1-R-AscI	A GGCGCGCCC TTCACTTGACTCCCCTC
	BSL1 promoter-F	GCTCCGCGGCCGCC ACTCAGTTGCATTGAATTTGAC
	BSL1 promoter-R	GCTCCGCGGCCGCC TGGAAACCACTTTACGGGTATAAATC
	BSL2 promoter-F	GCTCCGCGGCCGCC TTATCAAATTGTAGTCCATCCAAG
	BSL2 promoter-R	GCTCCGCGGCCGCC TATCAAAAAGCTTCAAAAGTGG
	Myr-F-NotI	GGCCGCCATGGGCAACAAATGTTGCAGCAAGCGACAGGATACC C
	Myr-R-XhoI	GAGCTGGTATCCTGTCGCTTGCTGCAACATTTGTTGCCCATGGC
	BSL2cds2NLS-R-AscI	AGGCGCGCCCTACCTTTCTCTTCTTTTTTGGATCTACCTTTCTCTTCTTTTTTGGATCagcagctgcCATCCAAGCCAGAGAACC
	BSL3cds2NLS-R-AscI	AGGCGCGCCCTACCTTTCTCTTCTTTTTTGGATCTACCTTTCTCTTCTTTTTTGGATCagcagctgcTATCCAAGCAAGAGAGC
	BSU1cds2NLS-R-AscI	AGGCGCGCCCTACCTTTCTCTTCTTTTTTGGATCTACCTTTCTCTTCTTTTTTGGATCAGCAGCTGCTTCACTTGACTCCCCTC
	BSL2-N587AA-R-AscI	A GGCGCGCCC GATTAGCGAAAATGTAGGTTTC

	BSL2-C-F-NotI	GCTCCGCGGCCGCC CGTGGTTGGAAGCCTCCTGTTC
Site-directed mutagenesis	MAPK6D218G-F	TCGAGTCACTTCTGAGAGTGGTTTCATGACTGAATATGTTG
	MAPK6D218G-R	CAACATATTCAGTCATGAAACCACTCTCAGAAGTGACTCGA
	MAPK6218GE222A-F	GAGAGTGGTTTCATGACTGCATATGTTGTCACGAGATGG
	MAPK6218GE222A-R	CCATCTCGTGACAACATATGCAGTCATGAAACCACTCTC
Protein fusion with GST	BSL2cds-F-EcoRI	CG GAATTC ATGGATGAAGATTCGTCTATGG
	BSL2cds-R-SalI	GC GTCGAC CATCCAAGCCAGAGAACC
