# Supplementary material for: Dichotomy of the BSL phosphatase signaling spatially regulates MAPK components in stomatal fate determination
Source: Nat Commun. 2022 May 4;13:2438. doi: 10.1038/s41467-022-30254-2 (PMC9068801; doi:10.1038/s41467-022-30254-2)
Supplement: Supplementary file 3 — Reporting Summary [file 41467_2022_30254_MOESM3_ESM.pdf]

nature portfolio

Corresponding author(s): DBPR-NCOMMS-21-46994A

Last updated by author(s): Apr 16, 2022

Reporting Summary

Nature Portfolio wishes to improve the reproducibility of the work that we publish. This form provides structure for consistency and transparency in reporting. For further information on Nature Portfolio policies, see our Editorial Policies and the Editorial Policy Checklist.

Statistics

For all statistical analyses, confirm that the following items are present in the figure legend, table legend, main text, or Methods section.

n/a	Confirmed	
☐	☒	The exact sample size (*n*) for each experimental group/condition, given as a discrete number and unit of measurement
☐	☒	A statement on whether measurements were taken from distinct samples or whether the same sample was measured repeatedly
☐	☒	The statistical test(s) used AND whether they are one- or two-sided<br>*Only common tests should be described solely by name; describe more complex techniques in the Methods section.*
☒	☐	A description of all covariates tested
☒	☐	A description of any assumptions or corrections, such as tests of normality and adjustment for multiple comparisons
☐	☒	A full description of the statistical parameters including central tendency (e.g. means) or other basic estimates (e.g. regression coefficient) AND variation (e.g. standard deviation) or associated estimates of uncertainty (e.g. confidence intervals)
☐	☒	For null hypothesis testing, the test statistic (e.g. *F*, *t*, *r*) with confidence intervals, effect sizes, degrees of freedom and *P* value noted<br>*Give P values as exact values whenever suitable.*
☒	☐	For Bayesian analysis, information on the choice of priors and Markov chain Monte Carlo settings
☒	☐	For hierarchical and complex designs, identification of the appropriate level for tests and full reporting of outcomes
☒	☐	Estimates of effect sizes (e.g. Cohen's *d*, Pearson's *r*), indicating how they were calculated

Our web collection on statistics for biologists contains articles on many of the points above.

Software and code

Policy information about availability of computer code

Data collection	Confocal images were captured by the Leica SP5 II microscope. Images were processed with Fiji (Image J) software (http://fiji.sc/Fiji)
Data analysis	Quantification and statistical analyses were performed using Fiji and GraphPad Prism 5.1. Histograms and plot boxes were generated by GraphPad Prism. Figure panels were assembled by Adobe Illustrator. Protein sequences were aligned with Clustal W.

For manuscripts utilizing custom algorithms or software that are central to the research but not yet described in published literature, software must be made available to editors and reviewers. We strongly encourage code deposition in a community repository (e.g. GitHub). See the Nature Portfolio guidelines for submitting code & software for further information.

Data

Policy information about availability of data

All manuscripts must include a data availability statement. This statement should provide the following information, where applicable:

- Accession codes, unique identifiers, or web links for publicly available datasets
- A description of any restrictions on data availability
- For clinical datasets or third party data, please ensure that the statement adheres to our policy

All data generated or analyzed during this study were included in this published article and supplementary files. Bio-reagents are available for research purpose upon request from the corresponding author under a material transfer agreement.

Field-specific reporting

Please select the one below that is the best fit for your research. If you are not sure, read the appropriate sections before making your selection.

☒ Life sciences ☐ Behavioural & social sciences ☐ Ecological, evolutionary & environmental sciences

For a reference copy of the document with all sections, see nature.com/documents/nr-reporting-summary-flat.pdf

Life sciences study design

All studies must disclose on these points even when the disclosure is negative.

Sample size	Sample sizes were determined by previous pilot experiments to be sufficient to achieve desired outcomes. Sample sizes are indicated in the Figures, legends and main text.
Data exclusions	No data were excluded from the study.
Replication	Numbers of replicates were stated in the legend and in the section of "Methods".
Randomization	Plant samples used in this study were genetically homogeneous and of the same age with same growth condition. Plants with the same genotype were randomly picked for experiments
Blinding	Blinding was not relevant to this study since all analyses were performed on homogeneous plant populations (see above).

Reporting for specific materials, systems and methods

We require information from authors about some types of materials, experimental systems and methods used in many studies. Here, indicate whether each material, system or method listed is relevant to your study. If you are not sure if a list item applies to your research, read the appropriate section before selecting a response.

Materials & experimental systems

n/a	Involved in the study	
☐	☒	Antibodies
☒	☐	Eukaryotic cell lines
☒	☐	Palaeontology and archaeology
☐	☒	Animals and other organisms
☒	☐	Human research participants
☒	☐	Clinical data
☒	☐	Dual use research of concern

Methods

n/a	Involved in the study	
☒	☐	ChIP-seq
☒	☐	Flow cytometry
☒	☐	MRI-based neuroimaging

Antibodies

Antibodies used	anti-Thiophosphate ester antibody Abcam Cat #ab92570<br>Anti-MPK6 Antibody Sigma-Aldrich Cat # A7104-200UL<br>Phospho-p44/42 MAPK Antibody Cell Signaling Technology Cat # 9101<br>GST (91G1) Rabbit mAb Cell Signaling Technology Cat # 2625<br>Monoclonal ANTI-FLAG M2 antibody(mouse) Sigma-Aldrich Cat # F3165<br>Anti-GFP Antibody Roche Cat#11814460001
Validation	All antibodies were validated by the suppliers by staining and western blot analysis.

Animals and other organisms

Policy information about studies involving animals; ARRIVE guidelines recommended for reporting animal research

Laboratory animals	no laboratory animals used in this study.
Wild animals	no wild animals used in this study.
Field-collected samples	no field-collected samples used in this study.
Ethics oversight	No ethical approval was required in this study.

Note that full information on the approval of the study protocol must also be provided in the manuscript.
